# p.Gln318X and p.Val281Leu as the Major Variants of *CYP21A2* Gene in Children with Idiopathic Premature Pubarche

**DOI:** 10.1155/2020/4329791

**Published:** 2020-05-15

**Authors:** Mahdieh Soveizi, Nejat Mahdieh, Aria Setoodeh, Fatemeh Sayarifard, Farzaneh Abbasi, Himangshu S. Bose, Bahareh Rabbani, Ali Rabbani

**Affiliations:** ^1^Rajaie Cardiovascular Medical and Research Center, Iran University of Medical Sciences, Tehran, Iran; ^2^Growth and Development Research Center, Tehran University of Medical Sciences, Tehran, Iran; ^3^Cardigenetic Research Center, Rajaie Cardiovascular Medical and Research Center, Iran University of Medical Sciences, Tehran, Iran; ^4^Children's Medical Center Hospital, Center of Excellence, Tehran University of Medical Sciences, Tehran, Iran; ^5^Biomedical Sciences, Mercer University School of Medicine, Savannah, GA, USA

## Abstract

Premature pubarche (PP) is the appearance of sexual hair in children before puberty. The PP phenotype may attribute to nonclassic congenital adrenal hyperplasia (NC-CAH). In this study, we investigated the role of *CYP21A2* gene variants in patients with PP in the Iranian population. Forty patients (13 males and 27 females), clinically diagnosed with PP, were analyzed for molecular testing of *CYP21A2* gene variants. Direct sequencing was performed for the samples. Also, gene dosage analysis was performed for the cases. Fourteen patients (35%) had a mutation of p.Gln318X and p.Val281Leu, out of which 10% had regulatory variants. Approximately 10% of the patients were homozygous (NC-CAH). 78.5% (11/14) of patients had trimodular RCCX of which 5 patients had two copies of *CYP21A1P* pseudogene. The prevalence of p.Val281Leu was higher than p.Gln318X in PP patients. In conclusion, *CYP21A2* variant detection has implications in the genetic diagnosis of PP phenotype. The genetic characterization of the *CYP21A2* gene is important for characterizing the variable phenotype of carriers and genetic counseling of PP and NC-CAH patients.

## 1. **Introduction**

Premature pubarche (PP) is presented by precocious appearance of sexual pubic hair before the age of 8 years in girls and 9 years in boys without any sign of puberty or virilization [1, 2]. The clinical symptoms usually include advanced bone maturation, increased growth velocity, presence of axillary hair, acne, oily hair, adult apocrine secretion which is known as premature adrenarche (PA). However, the appearance of pubic hair named as PP is the major symptom and half of the children with PA have pubic or axillary hair [1, 3]. Usually, PP is presented with major symptoms of nonclassical congenital adrenal hyperplasia (NC-CAH) [4]. It is diagnosed based on the exclusion of precocious puberty and NC-CAH [5]. Diagnosis and control treatments are important for the final height. Also, females may present more sympotms of hirsutism and irregular menstrual in older ages [5].

In precocious puberty, early maturation of the zona reticularis leads to an increased level of adrenal androgens seen during puberty [6], mainly dehydroepiandrosterone (DHEA) and its sulfate (DHEAS). Most children with PP have an early idiopathic androgen level [5]. Different diagnosis criteria lead to PP, including ACTH-stimulated test and 17-hydroxyprogesterone (17-OHP) levels [7, 8]. PP may attribute to 0–40% of NC-CAH [5, 9] in different ethnicities. NC-CAH, known as late-onset CAH (LOCAH), is mainly due to P450c21 (21-hydroxylase) deficiency inherited as autosomal recessive. It is mainly caused by mutations in the *CYP21A2* gene [10]. Defect in other genes encoding steroidogenic enzymes such as 3β-hydroxysteroid dehydrogenase (HSD3B2), 17 hydroxylase, 17, 20 lyase, cytochrome P450 oxidoreductase (POR), dehydroepiandrosterone (DHEA) [11, 12], sulfotransferase (SULT2A1) [13], and PAPS synthase 2 (PAPSS2) [14] can affect the biochemical pathway of the adrenal androgens [6].

More than 250 mutations have been described in the *CYP21A2* gene in the human gene mutation database (HGMD: http://www.hgmd.org). Common mutations of the *CYP21A2* gene are responsible for about 75% of mutations in Iranian populations [15].

Molecular testing is useful for screening patients with PP symptoms in children who are carriers; it also may be helpful for the management of future pregnancies. Early molecular detection, thus, is used for children who have not presented the symptoms yet.

Here, we investigated children with PP in the Iranian population to find out the effect of *CYP21A2* mutations in PP as well as genotype-phenotype characterization of the patients.

## 2. Materials and Methods

### 2.1. Clinical Evaluations

Forty unrelated children, including 27 females and 13 males clinically diagnosed with PP, referred to the Pediatric Center of Excellence and Growth and Development Research Center were enrolled in this study from 2015 to 2016. The clinical evaluation was performed by pediatric endocrinologists. This retrospective study was approved by the ethical committee of the Children's Medical Center Hospital, the Pediatric Center of Excellence in Tehran. Informed consent was taken from patients and the parents or guardian of the minors. Family history and clinical evaluations were documented for each patient. Biochemical examinations were also performed for the patients.

### 2.2. Genetic Testing

Five mL of peripheral blood were taken, and DNA was extracted using the salting-out protocol [16]. Coding, regulatory, exonic regions, and intronic boundaries of *CYP21A2* were amplified by specific primers [15, 17]. Direct sequencing was performed by ABI sequence Analyzer (ABI 3500, Applied BioSystems, US). Deletions/gene conversions and duplications were performed based on MLPA (MRC-Holland, Netherland) analysis for the patients.

Genetic analysis was based on reference gene NM_000500.7. Variants were named based on human genome variation nomenclature (HGVS). Segregation analysis was not performed due to the unavailability of the sample.

### 2.3. Multiple Sequence Alignment

Alignment with different species sequences was performed using UniProt multiple alignments (https://www.uniprot.org/). Conservation was investigated among different paralogs.

### 2.4. Functional and Structural Analysis

The protein sequence of CYP21A2 (UniProtKB/Swiss-Prot : P08686) was compared using the protein homology/analogy recognition engine (PHYRE2) for structural and functional analysis of the variants [18].

### 2.5. Interactome Analysis

Proteins' interactions were investigated to see the relation to other proteins of being causative in a systematic pathway by STRING 10 [19]. This is to investigate the interactions of the enzyme in the steroid biosynthesis pathway and, therefore the genes interactions in disease.

## 3. Results

### 3.1. Clinical Evaluations

The patients who were clinically diagnosed by a pediatric endocrinologist were enrolled in the study. Physical examinations confirmed PP. The clinical evaluation and biochemical analysis of genotype positive patients are presented in Table 1. Females included 71% (10) of the patients. The mean age of diagnosis was 6 y, 11 m ± 12.4 m for the patients. The presenting sign and symptoms mostly included pubic hair and axillary. No sign of breast bud in girls or an increase of testicular size in boys with *CYP21A2* gene mutation was found in the cases. Bone age was evaluated for each patient (Table 1) [20]. No sign of virilization and CAH was observed in the genotype positive patients. The bone age to chronological age ratio is also determined for the patients. The mean height of the positive cases was 129.14 ± 12.07 cm. In addition, the mean weight was 30.31 ± 8.3 Kg. The biochemical and hormonal analysis was performed for each patient for the clinical diagnosis.

### 3.2. Molecular Analysis

Totally, 35% (14 cases) of the patients showed *CYP21A2* variants. The patients were categorized based on *CYP21A2* genotype: (1) 65% (*n* = 26) of patients without *CYP21A2* mutation, (2) 15% (*n* = 6) of patients with a mutation in one allele *CYP21A2*, (3) 10% (*n* = 4 homozygotes and NC-CAH) of patients with two pathogenic alleles, and 2.5% (*n* = 1 compound heterozygote) and (4) 10% (*n* = 4) of patients with regulatory variants (Table 1).

Ten cases carried common *CYP21A2* variants at position p.Val281Leu (p.Val282Leu according to nomenclature) and p.Gln318Ter (p.Gln319Ter according to nomenclature), which accounted for 25% of cases. Three cases were male homozygous for p.Val281Leu, of which one had a family history of PP. Gene dosage analysis of these cases showed three copies of *CYP21A1P* pseudogene (duplication) and two copies of the *CYP21A2* gene (normal). This means that there is a duplication of a pseudogene. The homozygous regulatory variants carried two copies of the *CYP21A2* gene. Since a normal individual has two copies of the *CYP21A2* gene, this patient is considered normal with MLPA analysis.

Two cases were heterozygous for p.Val281Leu which carried two copies of *CYP21A1P* pseudogene (trimodular). In addition, four heterozygous cases for p.Gln318X had three copies of *CYP21A2* (duplicate) and one *CYP21A1P* pseudogene. Quantitative analysis of the compound heterozygous patient showed trimodular RXXC genotype for the patient. The patients' carrying heterozygous regulatory variants had three, two, and two copies of the *CYP21A2* gene, respectively. To note, segregation analysis of regulatory variants helps to distinguish the orientation of variants in each allele. Genotypes of the patients are shown in Table 1.

### 3.3. Protein Sequence Alignment

**Figure 1:**
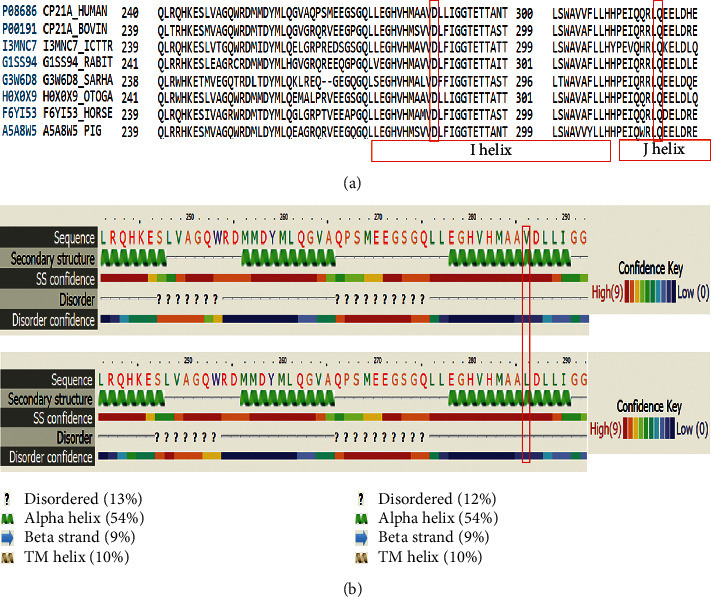
(a) Multiple amino acid alignment of CYP21A2 protein adapted from UniProt protein family members. Val282, as indicated in the box, is a highly conserved amino acid among different species. Also, Gln319 (known as Gln318) is depicted in the diagram. The determination of I-helix and J-helix (second structure) is shown in this figure within the box [21]. Panel (b) is a structural analysis based on Phyre2 software. The first line is amino acid sequence (UniProt P08686) of CYP21A2. The second line shows the secondary structure prediction, which is determined as alpha helix, with high confidence value (third line). Homology model was based on d3czha1 (Cytochrome P450) template with 100% confidence and the amino acid change in the fourth line (in comparison) is disordered which means it is not dynamic, with low confidence.

Val282 (named as Val281) and Gln319 (named as Gln318) were aligned among other species for the degree of conservation (Figure 1(a)). Both variants were highly conserved in the position; therefore, any change affects protein function at these positions.

### 3.4. Structural and Functional Analysis

Besides the numerous structural and functional studies performed in literature, we have performed in silico analysis to correlate the functional effect of common variants to variable expression of the phenotypes. Secondary structure analysis of Val282 was performed for missense mutation based on the d3czha1 (Cytochrome P450) template. It is a highly conserved alpha helix domain. Change in the amino acid caused disordered effect (Figure 1(b)). The severity of the structural change of the amino acid change is not very significant.

**Figure 2:**
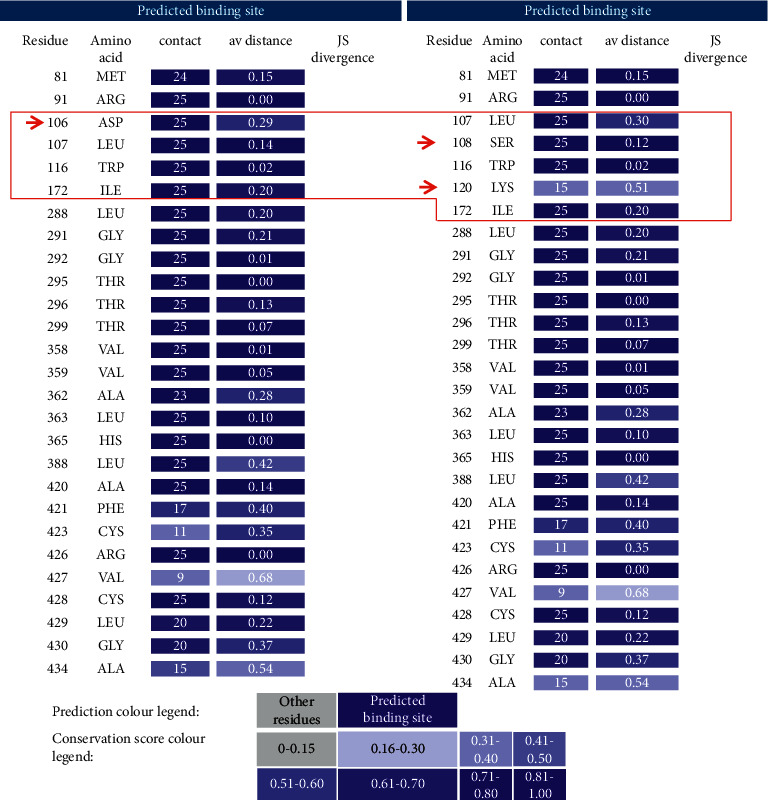
Binding sites of normal (left side) and mutant (right side) of p.Val281Leu in CYP21A2 protein predicted by 3DLigandSite server. Average distance ranges from 0–1.00 for each residue, which it was affected by this substitution as shown in red box; a new binding site, Lys120, is added based on this prediction.

Functional analysis of Val282 by 3DLigandSite server tool demonstrated a defect in binding sites with lowering the values and changing the binding sites (Figure 2). The binding sites score had a low level of significance (Figure 2), but a binding site at position Lys120 is added which may influence the enzyme activity of the 21-hydroxylase.

### 3.5. Interactome Analysis

**Figure 3:**
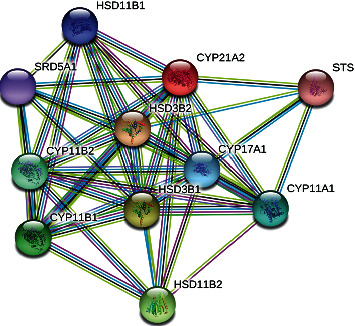
Protein-protein interactions of CYP21A2 protein depicted by STRING 10. HSD3B2 : hydroxy-delta-5-steroid dehydrogenase, 3 beta- and steroid delta-isomerase 2; HSD11B1 : hydroxysteroid (11-beta) dehydrogenase 1; CYP17A1 : cytochrome P450, family 17, subfamily A polypeptide 1; SRD5A1 : steroid-5-alpha-reductase, alpha polypeptide 1; STS : steroid sulfatase (microsomal), isozyme S.

As shown in the interaction of 21 hydroxylases *CYP21A2* is with hydroxyl-delta-5-steroid dehydrogenase, 3 beta and steroid delta-isomerase2 (*HSD3B2*); Cytochrome P450 family 17, subfamily A polypeptide 1, *CYP17A1*, cytochrome P450 family 11, subfamily B polypeptide 1 (*CYP11B1*), cytochrome P450 family 11, subfamily B polypeptide 2, *CYP11B2*, Steroid 5 alpha reductase alpha polypeptide *SRD5A1*, Steroid sulfatase isozyme S, *STS* (Figure 3). This protein-protein interaction shows that other gene defects in this interaction may cause phenotypic variability in the onset and severity of the disease.

## 4. Discussion

PP has an unknown etiology. Increased levels of adrenal androgens and hypersensitivity to steroid hormones are proposed to cause PP. Mutations of *CYP21A2* and *HSD3B2* genes have been described in PP patients [22–24]. The mechanism of disease is also still not understood completely, but 21-OH, 17-OH, and 17, 20-lyase and 3B-HSD are involved in the processes leading to puberty [6]. Therefore, the genetic analysis of one of the important genes involved in PP was investigated in our population. Previous studies have focused on the frequencies of *CYP21A2* mutations in patients with CAH in Iran [15]. In this study, 14 patients carried *CYP21A2* gene variants in PP patients. Interestingly, only two common variants, p.Gln318X (severe mutation) and p.Val281Leu (mild mutation), were responsible for presenting PP. In the study by Ghizzo *et al.*, the frequencies of these two common variants were also higher [25]. The frequencies of the heterozygous severe variant (p.Gln318X) were higher than the mild variant in the studied population, but the frequency of homozygous mild variant (p.Val281Leu) was higher, and no cases had a homozygous variant of p.Gln318X in this study. The question is why these two common mutations have shown the PP phenotype?

As known, p.Gln318X leads to salt wasting (SW) CAH in the homozygous state. Reports demonstrated the heterozygotes of p.Gln318X variant with only hirsute phenotype [26]. Gln319 is located on the J-helix of 21-hydroxylase [21] (Figure 1(a)). The loss of hydrophobic interactions of *CYP21A2* influences the protein stability and secondary structure, resulting in an inactive enzyme and SW CAH [27]. Structural stability in *CYP21A2* is maintained in the C-terminal by a set of hydrogen bonds between Gln481, Gln319 (J-helix), and the backbone of carbonyl oxygens of Leu446 and Gln447 (L-helix). A truncated chain (p.Gln318X) does not have the structural stability at C-terminal. As noted, some carriers do not show any symptoms throughout the years, but some heterozygous carriers show NC-CAH form [28, 29]. Previous studies in Iran showed patients carrying heterozygous p.Gln318X variant in NC-CAH cases [30]. We also have described the genotypic effect of a mutation in patients with different cardiomyopathies; as reported, a variant showed phenotypic variability in dilated and hypertrophic cardiomyopathies [31].

On the other hand, our series have indicated that the heterozygous p.Gln318X patients had an arrangement of three copies of the *CYP21A2* gene. The PP phenotype might be due to the normal production of the 21-hydroxylase. The trimodular organization of RCCX in chromosome 6 accounts for 14% of populations. The trimodular RCCX accounted for 71% of the genotype positive patients; therefore, copy number variations could influence the expression of the genotype.

Another reason is that a heterozygous variant showing an impaired/variable phenotype is influenced by other genes/variants [32]. Heterozygous nonsense variants prevent *CYP21A2* synthesis. Patients not presenting SW form demonstrate aldosterone synthesis, which means they are compatible with synthesizing the 21-hydroxylase enzyme [33] although studies demonstrated that the mRNA synthesis was decreased in vitro. It is suggested that mRNA downstream of Gln319 may not carry ribosomes that are susceptible to nucleases. However, epigenetic and nongenetic factors may influence the ability to synthesize aldosterone. Likewise, the gene controlling its biosynthesis differs from 21-hydroxylase [34].

As interactome analysis shows, other genes may interact with genes in steroid and adrenal biosynthesis; any change in their interaction may influence the phenotype.

The missense variant at p.Val281Leu causes 20–50% enzyme activity in NC-CAH. There are reports of this mutation in PP patients [25, 35]. In our study, 15% of the PP patients carried p.Val281Leu. In another study by Savas Erdeve *et al.,* the heterozygous patient showed PP [36]. It is reported that *CYP21A2* mutations increase the risk of hyperandrogenism, especially in carriers of p.Val281Leu than carriers of severe mutations [35, 37]. Residue Val282 is located on I-helix [21] in the *CYP21A2* gene in a restricted space. If leucine residue is substituted, the length of the chain increases and causes in steric clashes (influences the conformation and reactivity) in NC-CAH [27].

To explain, the structural analysis predicts the severity of phenotype but the zygosity of the variant and compound heterozygosity may exhibit different activity. Residue Val282 influences hydrophobicity in helix I if the interaction with helix H is disrupted, which causes SV phenotype [27]. Comparison of in vitro 17-OHP and progesterone [38] and *in silico* predicted activities (using a bovine based model and a human crystal) of ΔΔGs mutants of p.Val281Leu showed impaired heme incorporation to the enzyme [39]. Also, protein stability was not influenced [40]. *In silico* functional analysis showed that disruption by leucine substitution (Figure 2). Copy number variation also complicates the prediction of phenotype in p.Val281Leu as explained in our result.

Previous reports on patients carrying regulatory variants only reported the simple virilized form of CAH [41, 42], but variations of *CYP21A2* regulatory region were found in 10% of patients with PP (heterozygous cases: number 6, 7, and 8 and a homozygous case of 4).

*In silico* analysis of variant at position c.-447A>G also had a low pathogenicity level (Supplementary Table 1). In vitro and in vivo functional analysis is needed to prove the true pathogenicity of the variant. Transcription activity is influenced at different positions upstream of the *CYP21A2* gene [21]. Other variants in patients with PP are presented in Supplementary Table 1. *In silico* analysis showed the influence of these variants in comparison with other functionally evaluated variants at regulatory positions. This suggests the influence of the upstream variants, which may cause PP, in combination with other gene defects.

The prevalence of NC-CAH is between 1 in 1000 and 1 in 100 in the world [43, 44]. The frequency of heterozygotes also seems high. The carriers remain unidentified, and therefore genotyping helps the correct diagnosis of individuals [45]. ACTH-stimulated 17-OHP is frequently used for diagnosis, but this makes the prediction difficult due to overlapping concentrations [46]. The value of 17-OHP in this study specifies the diagnosis for hyperandrogenism and carrier state; it does not differentiate the NC form of 21-hydroxylase deficiency. As indicated, there is a wide range of 17-OHP values expressing the phenotype [47]. The frequency of NC-CAH seems to be higher in PP than the general population. It is mentioned that the frequency of CAH is high in Iran [17]. Therefore, it is estimated that the frequency of silent heterozygotes may be high; the mutant variant could be expressed in future generations with variable phenotypes. In our study, the frequency of heterozygosity in PP was 15%; the frequency of *CYP21A*2 heterozygous mutations is also high in PP patients in the world [35]. Genetic screening is recommended for newborns and affected families.

As known, the development of PP is associated with multiple variants, especially steroid enzyme pathway genes [6]. It is, therefore, recommended to check all of the bona fide genes such as *HSD3B, CYP11B1*, *POR*, *StAR*, *MRAP*, *MC2R, SULT2A1*, and *PAPSS2* in future studies [10], involving in steroidogenesis pathways in patients with PP phenotype. In addition, it should not be ignored that environmental factors and epigenetic influences in androgen expression and androgen receptor sensitivity affect the phenotype of patients [48].

There was not a straightforward relation between genotype and phenotype in our patients. There was no significant clinical characteristic between genotype positive (some considered as NC-CAH) and other PP patients in the study; we could not distinguish these two groups based on clinical characteristics [3, 5]; however, genetic testing provides information for the enzyme defect. Therefore, more patients should be investigated, and a detailed clinical characteristic is needed to correlate the genotypes of either heterozygotes or homozygotes to a phenotype. Also, segregation analysis could resolve the inheritance of specific alleles inherited to the child.

## 5. Conclusion

In conclusion, genetic testing of the *CYP21A2* gene and other related genes is recommended for individuals with PP. Genetic testing is advised for earlier management of patients for pubarche and final height. Affected females may be at risk of other puberty phenotypes. Therefore, individuals carrying the *CYP21A2* variant could be screened for the risk of having CAH offspring and other related phenotypes. The zygosity and copy number variations should be considered in variable expression of the phenotype.

## Figures and Tables

**Table 1:** The characteristics of patients with defined variants in the *CYP21A2* gene.

No	Gender	Referring age (years)	Family history	Clinical characteristics^*∗*^																			Nt. change	AA change	Location	Zygoisty/module
				Pubic hair stage	Bone age	Height/cm	Height SDS	Weight/Kg	Weigh SDS	BMI	BMI SDS	BA/CA	Axillary	F1 (μg/dL)	17-OHP (nmol/L)	T (ng/dL)	AE (ng/dL)	DHEA (μg/L)	ACTH-stimulation pg/mL	LH	FSH	E2				
1	F	6, 2 m	No	II	7	109	−1.369	17	−1.314	14.3	−0.665	1.12	I, adult odor	3	4.5	NA	3	400	1, 5, 2	0.6	1	2, 6, 10	c.844 G > T	p.Val281Leu	Exon7	Hom/tri
2	M	8, 6 m	+	II	12	140	1.725	46	3.169	23.46	2.992	1.41	II	19	17.5	—	1.7	284	17, 21, 25	0.6	1.5	—	c.844 G > T	p.Val281Leu	Exon7	Hom/tri
3	M	6, 10 m	No	I	6, 6 m	122	0.23	23	0.164	15.45	0.004	1.08	No	5	1.2	0.1	0.8	200	0.3, 1.5, 4	1	1.4	—	c.844 G > T	p.Val281Leu	Exon7	Hom/tri
4	M	5	No	II	7	108	-0.423	20	0.639	17.14	1.335	1.4	Adult odor	5	4	—	3	200	5	6	3	0.3, 1	c.-296T > C	—	5′UTR	Hom
																							c.-295A > C		5′UTR	Hom
																							c.-284A > G		5′UTR	Hom
																							c.-282T > G		5′UTR	Hom
																							c.-196T > C		5′UTR	Hom/bi
5	F	6, 11 m	No	No	9	130	1.774	31.5	2.064	18.64	1.594	1.3	II	3	13	—	7.9	—	—	0.3	1	—	c.955 C > T	p.Gln318X	Exon8	Het
																							c.844 G > T	p.Val281Leu	Exon7	Het/tri
6	F	7, 0 2 m	+	III	7, 10 m	126	0.588	42.2	3.286	26.58	3.759	1.09	No	10	0.9	0.15	0.5	350	NA	0.3	1	5	c.-296T > C	—	5′UTR	Het
																							c.-295A > C		5′UTR	Het
																							c.-284A > G		5′UTR	Het
																							c.-282T > G		5′UTR	Het
																							c.-196T > C		5′UTR	Het/bi
7	F	7, 3 m	No	II	7	124	0.316	24	0.269	15.61	0.088	0.96	II	11	1	0.2	1.5	200	3, 7, 10	0.9	1	5	c.-296T > C	—	5′UTR	Het
																							c.-295A > C		5′UTR	Het
																							c.-284A > G		5′UTR	Het
																							c.-196T > C		5′UTR	Het/bi
8	F	7	No	IV	11	143	2.932	33.6	1.732	16.43	0.417	1.42	II, adult odor	6	0.3	0.1	0.4	250	NA	0.2	2	8	c.-296T > C	—	5′UTR	Het
																							c.-447A > G		5′UTR	Het/tri
9	F	6, 5 m	No	II	6	122	0.758	22	0.231	14.78	−0.349	0.92	No	5	1	—	1.3	180	—	0.9	1	5	c.844 G > T	p.Val281Leu	Exon7	Het/tri
10	F	5, 6 m	No	II	9	123	2.195	31.5	3.047	20.82	2.707	1.64	Adult odor over height	6	3.05	—	1	1000	0.5, 5.0, 5.7	1	1	—	c.844 G > T	p.Val281Leu	Exon7	Het/tri
11	F	9	No	III	10	135	0.408	33.5	0.975	18.38	1.02	1.11	I	—	0.1	—	—	700	0.1, 1, 4	0.5	3.5	15	c.955 C > T	p.Gln318X	Exon8	Het/tri
12	F	8, 5 m	NA	II	11	140	1.852	35	1.605	17.86	0.957	1.3	II	—	0.1	—	—	—	0.1, 2, 9, 32	0.5	3.0	50	c.955 C > T	p.Gln318X	Exon8	Het/tri
13	F	7, 6 m	No	II	10, 9 m	146	3.962	35	2.223	16.42	0.49	1.43	I	NA	1	NA	2	300	NA	NA	NA	NA	c.955 C > T	p.Gln318X	Exon8	Het/tri
14	M	7	+	II	9	140	3.453	30	1.825	15.31	−0.128	1.28	I	10.5	0.2	0.01	0.9	300	0.2, 1.3, 2.3, 4.6	6.5	1.0	—	c.955 C > T	p.Gln318X	Exon8	Het/tri

T: testosterone (NR: 3–10 ng/dL); AE: androstenedione (NR: 0.28–1.75 ng/dL); E2: estradiol; F1: cortisol (NR: 5–23 *μ*g/dL); F: female; M: male; Het: heterozygote; Hom: homozygote; DHEA: dehydroepiandrosterone (NR: 3–83 *μ*g/L); ACTH: adrenocorticotropic hormone (NR: 25–100 pg/mL); 17-OHP: 17 *α*-hydroxyprogesterone (NR: 0.2–2.3 nmol/L); LH: luteinizing hormone; FSH: follicle-stimulating hormone; B : breast; Tes: testicular enlargement, *m* = months, NA = not available, BA/BC = bone age/bone chronological age. ^*∗*^According to human genome variation nomenclature, the naming is p.Gln319Ter, which is routinely used as p.Gln318Ter as previously named; and p.Val282Leu is named as p.Val281Leu.

## Data Availability

The data used to support the findings of this study are available from the corresponding author upon request.

## Supplementary Materials

Supplementary Materials In silico analysis of the upstream variants found in PP patients in this study.
