# Supplementary material for: p.Gln318X and p.Val281Leu as the Major Variants of CYP21A2 Gene in Children with Idiopathic Premature Pubarche
Source: Int J Endocrinol. 2020 May 15;2020:4329791. doi: 10.1155/2020/4329791 (PMC7355357; doi:10.1155/2020/4329791)
Supplement: Supplementary Materials — In silico analysis of the upstream variants found in PP patients in this study. [file 4329791.f1.docx]

**p.Gln318X and p.Val281Leu as the major variants of *CYP21A2* gene in children with idiopathic premature pubarche**

Supplementary table-*In silico* analysis of upstream variants found in PP patients in this study.

| Mutation | | Location | Rs# | Genotype | In silico/CADD PHRED | No. PP patients/zygosity | No. patients/ combination |
| --- | --- | --- | --- | --- | --- | --- | --- |
| DNA | Amino acid |  |  |  |  |  |  |
| c.-33G>A | - | 5'UTR | rs560258688 | G/A | 8.69 | 1/het | - |
| c.-536C>T | - | 5`UTR | rs6906026 | C/T | 2.129 | - | 2/c.-464het |
| c.-551A>G | - | 5`UTR | rs396458 | A/G | 2.845 | 2/het | 1/c.Q319X+c.-536het |
| c.-464G>C | - | 5'UTR | rs368531335 | G/C | 1.855 | 4/het | 2/c.-536het  1/c.-296het |
| c.-447A>G | - | 5'UTR |  | A/G | 4.833 | - | 1/c.-296het |
